# Applying Evolutionary Thinking to the Study of Emotion

**DOI:** 10.3390/bs3030388

**Published:** 2013-07-17

**Authors:** Glenn E. Weisfeld, Stefan M. M. Goetz

**Affiliations:** Department of Psychology, Wayne State University, Detroit, MI 48202, USA; E-Mail: ad4297@wayne.edu

**Keywords:** emotion, Cannon-Bard, limbic system, functional adaptation, neuroendocrinology, comparative psychology, elicitor, appraisal, affect, motivated behavior

## Abstract

This paper argues for invoking evolutionary, functional thinking in analyzing emotions. It suggests that the fitness needs of normal individuals be kept in mind when trying to understand emotional behavior. This point of view is elaborated in sections addressing these topics: defining emotion; applying comparative analysis to the study of emotions; focusing on the elicitors and resulting motivated behaviors mediated by the various affects; recognizing that not all emotions have prominent, distinct facial expressions; acknowledging all of the basic emotions and not just some exemplars; crediting the more sensible Cannon-Bard theory over James-Lange; recognizing the more ancient, fundamental role of the limbic system in emotion compared with that of the neocortex; and analyzing socio-emotional interactions as they occur naturally, not just individual emotional behavior studied under artificial conditions. Describing the various facets and neuroendocrine mechanisms of each basic emotion can provide a framework for understanding the normal and pathological development of each emotion. Such an inventory, or ethogram, would provide a comprehensive list of all of the observable behavioral tendencies of our species.

## 1. Introduction

The study of emotion is gaining increasing attention from psychologists. However, it sometimes suffers from insufficient application of evolutionary principles, like much of the rest of psychology. This paper suggests how evolutionary ideas can inform the study of emotion.

The paper begins by suggesting a comprehensive definition that embraces multiple aspects of an emotion, namely its elicitors and their appraisal, its affect, its overt behavior, and any nonverbal expressions and visceral changes. The next section argues for comparative and developmental analysis of human emotions, especially their expressions, functions and hormonal bases. This is followed by noting the importance of studying not just the perception of nonverbal expressions but also their other aspects, such as their effects on the behavior of the receiver and the molding of their form by natural selection. Next follows a plea for identifying all of the universal emotions, not just those with prominent facial expressions. A complete inventory of the basic emotions needs to include infantile emotions. The following section advocates for the Cannon-Bard theory and against overreliance on James-Lange. Discussed next are the roles of the limbic system and neocortex in emotional responding. The last section calls for more observational research on social emotional interactions. Emotion has mainly been studied under laboratory conditions one individual at a time. This gives short shrift to the dynamic, social aspects of many emotional encounters.

## 2. Need for Definitional Clarity

**Figure 1:**
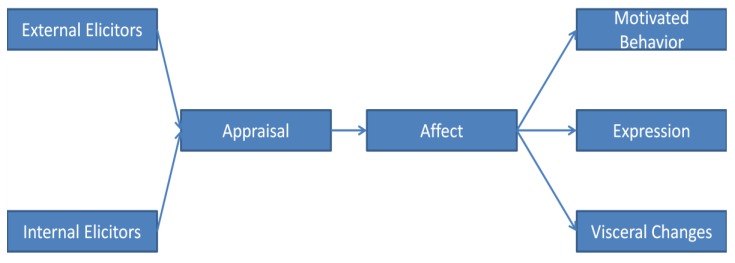
Basic schematic of constituents and their temporal sequence of emotion.

One serious problem is confusion about the definition of an emotion. Dozens of definitions are extant. General agreement exists that an emotion constitutes a behavioral complex, comprising several facets. People who study emotion usually focus on one or more of these facets, leading to disagreements on which to include in a definition of emotion. What are these facets (see Figure 1)?

Emotions have *elicitors*, although some definitions of emotion do not include this facet [1]. Regardless, emotions do have elicitors, whether or not elicitors are included in the definition. It makes no adaptive sense for an emotional response to occur haphazardly; emotions are elicited by internal and external changes that require a behavioral response.

These elicitors usually require some analysis, or *appraisal*. Some debate has surrounded the question of whether or not cognitive assessment always occurs [2,3]. Sometimes appraisal seems minimal. For example, a subject typically prefers familiar stimuli even if she does not recognize the stimuli as familiar. A rat will exhibit unlearned fear at the mere odor of a cat, responding to this elicitor, or releaser, with a fixed action pattern and without any deliberation. Similarly, in humans, the amygdala has been shown to respond to fearful faces before the visual cortex, indicating a lack of cognitive appraisal while still allowing for emotional discrimination [4]. The issue is whether or not cognition, meaning here conscious awareness, always occurs when an emotion is experienced. One might say that if an effect occurs, emotion occurs, since affect is arguably the most distinctive facet of an emotion. But does a conscious affect occur in the familiarity effect, if one is not aware of the stimulus being familiar? In any case, some appraisal does seem to occur in that an elicitor selectively triggers an emotional response, regardless of the extent of our awareness of this process. Perhaps these difficult questions of consciousness should be subordinated to the matter of functional explanation. Certainly many adaptive behaviors can be conscious or unconscious. A newly learned response initially may be conscious, such as movements in learning to drive a car, but can then become habitual and unconscious. It is easy to understand the adaptive value of being able to relegate habitual responses to unconsciousness so that more deliberative behaviors can occupy our full attention.

Most scholars include *affect* in their definition of emotion, and other categories of behavior seem not to involve affect, such as reflexes. A basic affect is a universal pleasant or unpleasant sensation—not a neutral one—that cannot be confused with other affects. Appraisal is the process by which the appropriate affect is activated by the elicitor. This allows the organism to activate an appropriate emotional response. Since we can experience more than one affect at a time, we somehow prioritize these affects so that we address the most pressing need first [5]. We manage to compare these affects quantitatively even though they differ qualitatively. Panksepp [6] and Pugh [7] speculated that this prioritization may occur in the midbrain, which receives affective input from most or all emotional modalities through the hypothalamus. Affective value is also attached to previously neutral stimuli so as to draw our attention to them and render them memorable. We need to pay more attention to emotionally salient stimuli than to neutral ones, because emotionally salient stimuli are generally relevant to fitness considerations [8]. Lastly, affects drive operant conditioning because rewards and punishments are affective experiences, and can act as unconditioned responses in classical conditioning.

An effect has fitness value only insofar as it prompts an adaptive behavior. A given affect leads to a characteristic observable behavioral tendency, or *motivated behavior.* Sometimes a particular affect can lead to a variety of motivated behaviors. For example, a frightened animal may freeze, flee, hide, or fight. For this reason, it is usually best to define basic emotions in terms of their affects rather than their motivated behaviors [6]. In addition, sometimes a motivated behavior can be prompted by different affects, such as eating either from hunger or the desire to be sociable. The importance of emotions in behavior is underscored by the observation that virtually all of our voluntary behavior occurs in response to affects.

Some but not all emotions have distinct evolved *nonverbal expressions*. The general function of these expressions is to influence the receiver to the sender’s benefit. For example, an angry expression can obviate the need for a costly physical altercation. Often it is claimed that expressions function to inform the receiver (e.g., [9]). But this claim constitutes a (largely discredited) group selection explanation, and does not explain the fact that some animal signals are deceptive.

Some but not all emotions have distinct patterns of *visceral adjustments*. These adjustments aid in performing the motivated behavior. However, if the motivated behavior does not entail vigorous exertion, these visceral changes may be minor and indistinct.

An emotion therefore includes elicitors, an effect, and a motivated behavioral tendency, and may also include a distinct expression and visceral changes. This definition would seem to embrace the factors examined in most of the research being conducted on emotion and motivation.

## 3. Need for Comparative, Functional Perspectives

Because emotions are behavioral complexes, they presumably took a long time to evolve. Therefore it is unlikely that any human emotions lack antecedents in ancestral species. Panksepp [6] argued that there are no uniquely human emotions. Yet most textbooks on emotion devote little attention to emotion in other species. Presumably, each basic human emotion can be traced to antecedent taxa and we can learn about it from comparative analysis of its facets. Comparative analysis is used to trace the phylogenetic origins of a given human emotion or facet of an emotion. For example, van Hooff [10] described similarities in the nonverbal facial expressions of various monkey, apes, and humans. He observed that the play face expression is so similar between chimpanzees and humans that each species responds appropriately to the expression in the other. Neuroscience comparisons can aid in discovering these continuities, especially since the limbic structures that mediate various aspects of emotion are highly conserved across vertebrates [11]. Relying on neuroscience data and behavioral observations can often allow us to identify continuities in particular emotions across the mammals [6]. Also helpful sometimes is reverse engineering: hypothesizing the existence of an emotion that would be necessary to address a particular fitness need [7].

Phylogenetic analysis can also reveal how an emotion has evolved within a taxon. For example, the “rage reaction” in mammals can be elicited by attack or restraint. This may be the original, most general elicitor of this emotion. In some mammals, the vehement aggression of rage can also be evoked by incursions into the animal’s territory, challenges to its rank, or threats to its young. In humans, anger can be precipitated by other forms of harm inflicted by another, such as violations of social norms (see below). Like mammals in general, human infants typically exhibit rage when restrained. Later they are angered by insults and other norm violations. This developmental progression may reflect operation of the biogenetic law, which is generally valid even though the effects of heterochrony can violate it [12].

Phylogenetic and ontogenetic analyses of an emotion, starting first by recognizing its universality, can reveal its original function and how the emotion may have been modified by later selection pressure. For example, the emotion of pride and shame has been studied comparatively by Tracy, Shariff, and Cheng [13]. Tracy and Robins [14] demonstrated that the bodily expression of this emotion in humans occurs across cultures and presumably is universal. As Darwin and many others have remarked, the bodily expression of pride and shame parallel the dominance and submission displays of other primates. However, additional parallels, or homologies, between dominance behavior in other species and competitive behavior in humans are seldom noted [15] even though dominance behavior is observed in hens [16] and even crickets [17]. Analysis of dominance behavior in humans and other species reveals that this behavior functions to motivate the individual to challenge rivals in order to advance in rank and gain prerogatives, and to challenge them judiciously. Dominance and submission displays, as well as recall of previous outcomes, reduce the frequency of conflict by making outcomes predictable. Because dominant individuals expect to succeed, they exhibit less fear and greater gregariousness and competition seeking. Functional analysis of pride and shame in terms of concrete fitness benefits such as mating opportunities can be contrasted with some extant psychological explanations. The latter often are phrased in terms of communicating status alone, or informing oneself of one’s place in the social order.

A comparative perspective would also suggest that distinctions between shame, guilt, and embarrassment are only semantic or contextual since all of these involve the same affect [7,18,19], expressions (Ekman in [20], p. 391), neural bases, developmental onset, yielding of resources, hormonal changes, and social withdrawal [15]. Guilt and shame may arise in different contexts, but the emotion seems to be fundamentally the same. Various contextual distinctions have been proposed between the so-called negative self-conscious emotions”; for example, Tangney, Miller, Flicker, and Barlow [21] have asserted that shame occurs in public and guilt in private. However, respondents disagree considerably about these distinctions within and across cultures [22,23,24]. We have argued, however, that embarrassment includes an element of humor appreciation [25], making embarrassment an emotional blend and not basic.

Hormones are an important factor in emotional behavior and, since most hormones are phylogenetically ancient, they can be studied comparatively. Some emotions induce secretion of hormones that enhance performance of the motivated behavior. Fear, anger, and various other stressors raise corticosteroids and epinephrine, leading to adaptive metabolic and visceral adjustments.

Hormones can also elicit various emotional changes. Melatonin induces drowsiness. Leptin reduces hunger. Various hormones of pregnancy and lactation promote maternal behavior. Vasopressin has been associated with defense of the young in monogamous male voles [26]. Prolactin rises in pair bonding males, including men, with a pregnant mate, and is correlated with men’s nurturant tendencies [27]. Indeed, in mice, prolactin has been shown to be a key mediator in paternal offspring recognition [28]. Oxytocin in women and vasopressin in men are associated with marital satisfaction [29]. Testosterone increases libido, reduces fear [30], and enhances self-esteem and self-confidence [31]. The sex difference in testosterone secretion might help explain sex differences in fear, competitiveness, and depression [32].

Sometimes a hormone is secreted in response to some emotional experience and then induces further motivated actions. Competitive success raises testosterone in men, promoting continued openness to challenges under auspicious conditions, leading to further competitive behavior toward a novel opponent [33]. Competitive failure increases hypothalamic-pituitary-adrenocortical (HPA) axis activity and fearfulness, and avoidance of competition [34,35]. Various positive social interactions, primordially nursing, increase oxytocin, which in turn promotes social bonding while reducing fear [36] and increasing trust [37]. To the extent that a social bond is protective, reduced fear is appropriate. Oxytocin also increases sensitivity to facial expressions [38], which would be desirable for a mother with an infant. In women, vasopressin increased smiling and friendliness, but it increased frowning in men [39]. This sex difference is consistent with social psychological research indicating that men who are insulted tend to retaliate in anger, whereas women react with friendliness and appeasement [40]. This study calls to mind research on female mammals’ (including women’s) “tend and befriend” reaction to threat, mediated by oxytocin: females protect their young and seek protection from others, whereas males prepare to fight [41].

## 4. Need to Go beyond Studying Perception of Expressions

One of the greatest successes in the study of emotion may have distorted the study of emotion. Darwin’s *Expression of the Emotions in Man and Animals* is probably the greatest book on emotion and perhaps even in developmental psychology and human ethology [20]. Darwin employed comparative, cross-cultural, physiological, and developmental methods to identify basic human expressions, thus establishing these psychological tools for identifying behaviors with an evolved basis. About a century later, Ekman and Friesen extended Darwin’s cross-cultural research to identify some basic expressions further [42]. This work has inspired a great deal of research on perception of the six initial facial expressions identified by Ekman and Friesen as universal. But other facets of emotion have been neglected. For example, little research has been conducted on elicitors of an emotional expression, behavioral reactions to one’s affect, and, importantly, behavioral reactions to perceiving another person’s expression. It has been easy to study perception of emotional expressions in the laboratory; it is much harder and less common to observe behavioral reactions under natural conditions.

One particular need is to explore the evolution of various expressions. Darwin pioneered analysis of the form of various expressions: why a given expression takes the form that it does. He proposed three principles about this. These principles are rather neglected these days, but they can usefully be recast in modern ethological terms.

His *principle of direct action of the nervous system* refers to observable manifestations of autonomic nervous system changes. These include dilation of the pupils, trembling due to priming of the muscles, and pallor from diversion of blood from the skin, all sympathetic division changes. Penile erection, mediated by the parasympathetic division, might also be cited. Because these changes are observable, they reveal the person’s affect and hence constitute emotional expressions.

Darwin’s *principle of serviceable associated habits* would be regarded by ethologists as observable intention movements (Ekman commentary in [20], p. 54). Examples include preparation to dodge or flee in the startle response, protecting the body from a blow when flexing the trunk in submission, and opening the mouth in preparation for crying out in fear.

Darwin’s *principle of antithesis* refers to the fact that emotional expressions often come in pairs that take opposite forms. For example, pride (erect posture and direct gaze) and shame (slumped posture and gaze avoidance) are expressed in antithetical ways, as are happiness and sadness, and the raised eyebrows and facial pallor of fear are diametrically opposed to the lowered eyebrows and facial flushing of anger. A newly evolving expression sometimes takes a form opposite that of an antithetical emotion in order to induce the opposite behavioral reaction in the receiver. For example, if lowered brows originally indicated anger, perhaps the opposite expression of raised eyebrows evolved to indicate the opposite emotion, fear. The receiver would then interpret raised brows as meaning the absence of intention to attack.

Another factor in shaping emotional expressions, of course, is phylogeny. As mentioned above, van Hooff pioneered study of homologies in facial expressions in primates, including humans [10]. This basic work by Darwin and other ethologists needs to be pursued further.

## 5. Need to Recognize All Basic Human Emotions

Another unfortunate consequence of Ekman’s truly great work is that other emotions have been neglected. These neglected emotions include those for which facial expression is minimal but bodily expression is prominent—pride and shame, for example. Other neglected emotions are expressed vocally more than facially, such as pain, humor appreciation, and sexual pleasure. Still other emotions have no specific expression, such as love, and so have also received short shrift in textbooks. Many of the so-called motives, or drives, lack a distinct facial expression, such as hunger, thirst, sex, and drowsiness. Since these motives possess affects and other emotional facets, why not include them as emotions along with fear and anger? Doing so would provide more consistency with animal research, since animal ethologists do not distinguish between “motivated” behaviors such as feeding and “emotional” behaviors such as defense. Ekman himself, after initially insisting that a basic emotion must have a distinct facial expression, later acknowledged that many basic emotions lack a facial expression [43]. Nonetheless, the majority of studies of emotions use Ekman and Friesen’s Big Six and no other emotions, even when the topic is not expressions.

For the sake of completeness, it would seem important to identify all of the basic, universal human emotions. Yet most textbooks on emotion focus on only a few supposedly representative emotions, the evolutionist deCatanzaro being an exception [44]. Speculations about the basic human emotions seem to have fallen out of favor. A comprehensive human ethogram would list and describe all of the basic observable whole-body behaviors observed in all cultures. Such a model would include all classes of voluntary behaviors and their respective functions, and so would provide a framework for the study of human behavior. The basic human emotions can be identified by applying the research tests pioneered by Darwin: is the emotion universal, does it have phylogenetic antecedents, does it develop in the absence of opportunities for learning it, is it mediated by a specific physiological mechanism, and is it stereotypic in form [20]. This model could potentially include developmental changes in emotion, quantitative sex differences, and individual differences, including pathological conditions [45].

What are these neglected emotions? If we define a basic emotion as a universal behavior pattern with elicitors, a distinct affect, and a particular behavioral tendency, many others qualify besides the Big Six. Through the years, evolutionarily inclined theorists have included tactile pain and pleasure, hunger, thirst, disgust, sexual feelings, loneliness and love, interest and play, humor appreciation, pride and shame, fear, and anger. Less commonly mentioned are drowsiness, air hunger, excretory sensations, fatigue, and esthetic emotions such as beauty appreciation and music appreciation—noise avoidance. (The specificity of the behavioral tendencies prompted by the esthetic emotions is dubious; only approach and investigation characterize them.) Other guides to identifying basic emotions include a distinct expression, distinct representation in the limbic system, and presence in related species. It seems necessary to postulate the existence of a given emotion if the absence of the related motivated behavior would reduce the animal’s fitness. In other words, the complement of emotions of an animal constitutes its repertoire of essential observable behaviors [7].

One problem in listing basic emotions is that most theorists have focused on adult emotions. But emotions change qualitatively and quantitatively over development, and these developmental changes can be analyzed functionally. Infants have a strong need for contact comfort and derive pleasure from being rocked, thus inclining them to seek to be carried about by caregivers. Children and mammals generally are very curious, inclining them to learn while young enough to profit from their skills for a long time. Adolescence brings notable changes in nurturance, competitiveness, and libido, consistent with the onset of reproductive capability. Developmental psychology focuses on cognitive and linguistic changes in development, but emotional changes are also important.

## 6. Need to Credit the Cannon-Bard Theory

Many textbooks continue to endorse the James-Lange position [46,47] that affects occur *after* the motivated behavior and its accompanying visceral changes. But James-Lange has numerous flaws. Presumably, discrete affects evolved to direct adaptive motivated behaviors; what adaptive function would be served by informing the brain of what it should do after it has already acted? In James’ famous example, why would running from a bear be necessary to experience fear—if one froze in terror, would one not be afraid? If we start running spontaneously, will be become afraid? Engaging in various forms of exercise with its accompanying visceral changes does not elicit particular affects. Rolls argued, further, that it is not parsimonious for appraisal of a stimulus to have to await feedback from the periphery [48].

Yes, one has to appraise the situation to know that one must flee, but this appraisal immediately triggers the appropriate affect. Of course, someone might not reflect much on how afraid he was until he was safe; his attention would initially be directed at executing the escape response. After reaching safety, one might experience strong and prolonged fear in order to register the memory of events to be avoided in the future. But while fleeing, surely he was afraid, if only momentarily. His fear is what made his heart race; the James-Lange alternative would be that our appraisal of the situation dispassionately causes us to exhibit the appropriate set of visceral adjustments. Why is it only emotionally salient stimuli that elicit these visceral changes, if appraisal of the stimuli is dispassionate? Do we learn how to adjust our viscera for each appraised situation through our largely unconscious autonomic nervous system?

The James-Lange notion that visceral feedback dictates affects runs counter to the fact that, during an emotional episode, “The main function of the autonomic and endocrine changes is to bring various bodily reactions in line with behavioral/physiological demands of each emotional system” ([49], p. 258). Obviously, these visceral adjustments had to evolve later than the emotional behaviors that they facilitate, so emotional responses probably do not depend on them. To be sure, bodily feedback initiates some affects, such as low blood glucose or stomach contractions eliciting hunger. But hunger is not triggered *following* initiation of feeding or visceral adjustments to food intake; more on this below.

Cannon [50] and Bard [51] mounted convincing evidence for their contrary view that affect precedes action. Much of the feedback from the viscera—which is not highly specific for particular emotions [52]—takes several seconds to travel from and back to the brain—way too long to wait to be afraid. Even with greatly diminished visceral feedback, as in spinally transected patients and experimental animals, affective self-reports and behavioral responses are appropriate for the situation [53], even if affect is sometimes blunted—understandably in patients prone to depression [54].

Confusion about the role of visceral feedback may stem in part from the fact that we localize many affective sensations in the viscera or skin, such as tactile sensations, visceral pain, sexual pleasure, hunger pangs, dry mouth, and muscular fatigue. That is, emotional elicitors are often internal. Pugh described the affective events occurring in addressing a given emotional need [7]. Initially we experience what he called *antecedent values*, or affects, that prompt us to address the particular emotional need. For example, we experience hunger that prompts feeding, and this affect is localized in the stomach to some extent. As we perform the appropriate motivated behavior, we are guided by feedback that Pugh called *concurrent values*. For example, we select different foods as we consume a meal. Again, this affective stream is localized in the tongue, palate and stomach. When we finally have fulfilled the need, having performed the consummatory response, we experience a generally-positive *trailing value*, again localized in the stomach in this example. Most antecedent values are negative in valence, inclining us to correct some internal need or address some external challenge. But some antecedent values are positive, causing us to approach a beneficial elicitor, such as an interesting stimulus or potential mate. James-Lange claims that visceral and dermal affective sensations occur after the motivated behavior has begun, but the reverse seems to be true. Once the successful motivated action begins to occur and once it has been completed, the affective state (first concurrent and then trailing) generally becomes more positive. For example, we solve some problem and thereby satisfy our curiosity, or we have a sexual encounter. This usually leads to cessation of the motivated behavior. Again, affect influences motivated behavior, not the reverse.

The facial feedback hypothesis is also invoked in support of James-Lange. Indeed, tricking subjects into assuming a particular facial expression can induce or enhance the corresponding affect [55,56]. However, these effects are dubious, with many failures to replicate [57], and might be explained by classical conditioning through repeatedly experiencing the affect when exhibiting the expression. A patient with facial paralysis due to Guillain-Barré syndrome reported normal affective experience [58], and patients with facial paralysis due to Möbius syndrome experience affects and recognize facial expressions normally [59]. Similarly, Parkinson’s disease patients’ experience of emotions appears to be unimpaired despite of their ability to spontaneously express facially displayed emotions [60]. Facial and visceral feedback might intensify affects by means of conditioning, but they do not seem to be necessary for inducing the appropriate affect in the first place. In his later writings, James even disavowed his earlier claim that feedback induces the appropriate affect such as fear of a bear [61].

Recent evidence against James-Lange comes from LeDoux’s work on the mediation of emotional responsiveness in mammals such as fear behavior in the rat [62]. A mammal receives emotionally salient information through sensory systems that activate the thalamus. There a quick appraisal of the information takes place. If the stimulus constitutes a releaser of, say, fear, the animal exhibits the panoply of fearful behaviors. The central nucleus of the amygdala orchestrates the various facets of the emotional response. The central grey (midbrain limbic area) activates the overt behavior of freezing, and the hypothalamus initiates visceral adjustments such as increased heart rate and the expression of piloreection. But the effect of fear comes first: the amygdala, being affectively sensitive, presumably registers fear before these behavioral and visceral events have occurred. LeDoux’s model does not include feedback from the voluntary muscles, viscera, or display elements to the limbic system. Simply put, appraisal of an elicitor as emotionally salient directly activates limbic structures that have been shown by brain stimulation and lesion studies in humans to be sensitive to various specific affective experiences.

The neocortex is indeed capable of modifying emotional responses, but the basic vertebrate emotional system is limbic (cf. [63]). When the auditory cortex is lesioned in rabbits, the animal will still respond fully to appropriate fear releasers or conditioned stimuli. With the auditory cortex intact, the rabbit makes finer discriminations of these stimuli [64]. For example, it may not exhibit fear of a tone that differs from the tone it has been conditioned to fear. And of course the mammalian neocortex allows finer motoric responses. So the neocortex only refines emotional behavior but is not the basic mechanism, which is limbic. Appropriate emotional responding in all its manifestations—motivated behavior, visceral changes, and expressions—can occur in the absence of appraisal by the neocortex.

Another theory in the James-Lange tradition is the somatic marker hypothesis popularized by Damasio [65]. Damasio claimed that decisions are made by considering the increase in skin conductance (sympathetic arousal) that accompanies contemplating various response options. Subjects with damage to the ventromedial prefrontal cortex experienced no anticipatory skin conductance changes and exhibited poor decision making on the Iowa Gambling Task [65].

Dunn, Dalgleish, and Lawrence critiqued the theory [66]. They argued that successful gambling strategies seem to be consciously comprehended rapidly, leading to differences in anticipatory changes in skin conductance and thus reversing the direction of causality [67]. An alternative explanation for the somatic marker findings is that these patients suffer from reduced motivation to succeed at the task; apathy is a symptom of ventromedial prefrontal cortex impairment [68]. These prefrontal patients also exhibit future discounting [69], which would likewise impair performance; prefrontal lesions characteristically reduce delaying rewards. These James-Lange variants are also weakened by research on patients with defects in autonomic functioning. Six patients with pure autonomic failure actually performed better than controls on the Iowa gambling task [70].

In favor of the somatic marker hypothesis, decision making does seem to involve the ventromedial prefrontal cortex, amygdala, insula, and somatosensory cortex. These areas receive peripheral feedback directly or indirectly. However, it is equally plausible that the affective consequences of various response options are represented by activation of affectively sensitive areas such as these, so that the respondent can contemplate these anticipated emotional consequences when deciding on a course of action. Merely imagining carrying out various courses of action would not allow one to choose among them; imagining their respective affective payoffs is necessary. Panksepp suggested that peripheral feedback plays some role in generating different affects but that other inputs also are involved [71].

## 7. Need to Recognize the Central Role of the Limbic System in Emotion

Liotti and Tucker referred to “...the current fascination of neuroscientists for ‘cognitive’ models of emotion, leading sometimes to frankly excessive ‘cortical chauvinism ([72], p. 408).’” The overemphasis of the role of the neocortex in emotion was aided by Schachter and Singer [73]. They administered epinephrine to subjects who reported feeling “as if” experiencing an effect—but not actually experiencing an effect. Subjects who were placed in situations in which a particular emotion was appropriate, such as observing an anxious confederate, resulted in reports of that emotion. This research, although fraught with methodological problems, suggested that the context can influence emotional experience, a rather trivial conclusion. Current researchers have extended this notion of the relativity of emotion to social constructivist positions that call into question the very idea of discrete emotions with different and specific neural representations [74]. Some of these formulations have eschewed discrete emotions theory in favor of analysis of particular emotions into dimensions such as intensity and valence. Surely emotions can vary in intensity and valence, but they also vary qualitatively. For example, fear and anger are both negative and can be intense.

It makes little functional sense to suppose that identifying and addressing particular adaptive needs was left entirely to the vagaries of cognitive interpretation and experience rather than to specific, localized behavioral mechanisms. Specific mechanisms that address basic adaptive needs such as nutrition, respiration, excretion, defense, and reproduction were already differentiated in protozoans. Dethier’s research on the blowfly illustrates the specificity of neural systems for feeding and other motivated behaviors in an invertebrate [75]. Humans share a great many genes with even invertebrates, including genes for basic brain structure [76]. In reptiles which lack a neocortex and have limited cognitive abilities, emotional systems for flight, attack, feeding, *etc*. exist. Quite a bit of continuity exists in specific emotional behaviors in the vertebrates and even more in mammals [6]. Homologies in the expression of particular emotions between humans and simians have been demonstrated, beginning with Darwin [20]; if an expression is universal and evolved and associated with a particular limbic structure, why wouldn’t the associated affect be evolved and localized also?

Lindquist *et al*. reported a meta-analysis of neuroimaging studies indicating that individual emotions are not localized in particular brain areas [74]. Mapping a particular emotion onto a particular brain structure is complicated by several factors. When an emotional experience is evoked experimentally, many structures throughout the brain are activated, including the neocortical structures. Some of these structures may be shared by multiple emotions. The same brain structure may mediate appraisal of elicitors of various emotions, and another structure may mediate performance of various motivated behaviors. For example, the visual system is involved in seeing a food item and seeing an enemy, and the motor cortex may be activated when we eat and when we seize a weapon. In other words, the brain structures that mediate some facets of emotions may overlap. For example, disgust activates the anterior insula but also the amygdala. The amygdala may be activated because it attaches positive and negative valence to previously neutral stimuli, such as a disgusting image.

Nevertheless, some meta-analyses of neuroimaging studies have succeeded in localizing particular emotions [77,78,79]. These results have generally agreed with analyses based on lesion studies of humans and other species [80], providing convergent evidence for these conclusions. In the non-mammalian vertebrate classes, the execution of specific emotional behaviors is left mainly to the limbic system [11], and motivated actions tend to be more rigid, or stereotypic, than in mammals. Many vertebrate emotional responses consist of evolved, fixed action patterns in response to releasing stimuli. Therefore, localization of emotions in these species may be easier than in mammals, and especially than in the flexibly behaving primates such as humans.

What emotions have been fairly well localized? Neuroimaging studies have indicated that the affect and expression of disgust are localized in the insula, and fear and its expression in the amygdala. Ability to perceive fear expressions is also localized in the amygdala. Granted, additional affects can also be localized in each of these structures, but surely some specificity exists, rather than affective representation varying randomly. Other affects also seem to be somewhat localized, such as amorousness in the anterior cingulate gyrus [81], hunger in the lateral hypothalamus [82], and drowsiness in rostral regions of the hypothalamus expressing melatonin receptors [83]. More precision would doubtless be possible in localizing affects were it not for technical difficulties. For example, more precise localization might be possible if researchers could specify which of the 15 amygdalar nuclei they were studying. Also, often one experiences a blend of emotions, thereby complicating attempts at localization. For example, performing worse than someone of lower rank than oneself, or social rejection, activates the anterior insula—such an abject failure may be disgusting as well as shameful [84]. Then too, some commonly used terms for affects may be too vague for the precision of the brain, such as happiness and sadness, and therefore not readily localized.

Further indications of the localization of affects come from brain stimulation studies. Mammals possess reward and punishment brain areas [85] that are almost exclusively limbic [86]. These structures are generally homologous to human structures that, when stimulated in conscious neurosurgical patients, elicit reports of specific affects (e.g., [87] on anger; [88] on fear; [89,90] on humor appreciation). One patient with bilateral amygdala damage due to Urbach-Wiethe disease was impervious to the effect of fear—except for fear of anoxia, indicting a separate mechanism for that affect [91]. Evidence for specific affects arising during localized psychomotor epileptic seizures also abounds [88,92].

The issue of localization of emotions can also be considered from a developmental perspective. Any animal has to eat as soon as it is born or hatched, and modifies this primordial feeding responsive with experience. Infant emotions, such as hunger and fear, unfold through epigenetic programs according to a precise, universal timetable [93,94,95]. Disgust is expressed even in newborns. These facts support discrete emotions theory. A human infant would die if not equipped with subcortical behavioral mechanisms for communicating its specific and fluctuating emotional needs to caregivers without relying on learning or language. The idea that infantile emotions begin as undifferentiated positive and negative emotions, *i.e.*, distress and contentment, and only later become more specific, would have been maladaptive. Evidence exists that even at six months of age infants react differently and appropriately to fearful stimuli and to anger-inducing stimuli [96]. Several other emotions emerge by nine months of age [95].

Of course, the huge neocortex, comprising about 90% of brain volume, is there for a reason. It works hand in glove with the limbic system in refining emotional behavior. For example, children’s cognitive development, mediated by the neocortex, allows them to understand what behaviors are permissible, forbidden, and demanded. As Egas and Arno put it, decisions to punish misbehavior “come from an amalgam of emotional response and cognitive cost-impact analysis” ([97], p. 871).

## 8. Need for More Functional Analysis of Social Interactions

Trivers pioneered analysis of the function of guilt, moral anger, gratitude, and sympathy in reciprocal altruism [98]. He explained how these emotions enhance individual fitness by promoting the exchange of favors. One might extend this analysis. For example, pride may function, along with sympathy, to incline one to aid others, thereby resulting in incurring obligations and gaining resources from the recipient [99]. Making a charitable donation [100] or being cooperative [101] and presumably experiencing pride can activate the pleasure-mediating nucleus accumbens. Performing a favor raises one’s social status and inclines one to accept compensation, whereas receiving a favor is somewhat demeaning and inclines one to compensate the donor. Similarly, dominant animals strive to succeed in competition, thereby gaining resources yielded by subordinates. Equity is maintained between successful individuals—those who succeed in competition for social status by their accomplishments—and subordinates, in that rewards are proportional to accomplishments. We seek to maintain or restore equity and violations of equity are disconcerting. This might explain why receiving excessive praise is embarrassing, why people sometimes decline favors if unable to return them later, why transplant recipients often feel guilty, and why subjects sometimes punish misbehavior at some cost to themselves (which activates the reward-mediating dorsal striatum—[102]). We are uncomfortable with violations of equity, even if we benefit materially from them, and we are gratified by restoration of equity.

These transactional scenarios might be analyzed in more detail. For example, if a social norm is violated (not just a failure to return a favor but any violation of a social norm), the victim typically experiences anger [103,104]. This affect may result in an angry (threat) expression, withholding of resources, or actual attack. In any case, the target individual typically reacts with submission (or shame) and relinquishes resources—either by yielding them or promising to do so by apologizing. This restores the equity, or social homeostasis, prevailing initially. By making restitution, the target individual neutralizes his shame, just as the offended party has his anger appeased by this appeasement display. This interpretation is consistent with the notion that anger is triggered not generally by frustration, or blocking of one’s goal, but more specifically by a willful violation of a social norm [105,106]. It makes logical sense that restitution—correction of the violation—would neutralize anger and even be pleasurable: witnessing unfair individuals being punished activated the nucleus accumbens [107]. Similarly, a convincing apology or explanation by a third party will dissipate a victim’s anger [108] as will punishment of the violator by a third party [40]. Aggression redirected at an innocent party or inanimate object will not reduce the rise in systolic blood pressure associated with anger, presumably because these measures do not correct the injustice.

To add a comparative perspective to this idea of defense of social norms: in many social species violation of an animal’s territory, dominance claims, or possession norms can trigger a “rage reaction” that in effect punishes transgressors. In neurological terms, registering one’s obligations, *i.e.*, guilt [109] and embarrassment [110], involves the orbitofrontal part of the prefrontal cortex. Patients with ventromedial prefrontal cortical lesions behaved more selfishly than controls [111]. Comprehension of social norms and consideration of questions of fairness likewise activate areas of the prefrontal cortex [112].

Another example of how socioemotional interactions might be analyzed concerns humor. The humorist tells a joke, and the listener appreciates the joke and laughs. This expression implies an obligation on the part of the listener, who is inclined to compensate the humorist in some way. Perhaps the listener will pay a professional comedian, or offer jokes of his own, or provide some other compensation such as invitations to future social events. Similarly, other artistic performances are applauded and rewarded [113]. More tangibly, the content of jokes may be of some informational value to the listener, especially in illustrating gaffes to avoid [114]. Receiving this edifying information requires compensation or it would not continue to be offered.

As a last example, consider parental behavior. The parent and offspring must establish a mutual bond, or attachment, but this is not sufficient. The parent must be motivated to provide appropriate care to the offspring. How does this happen? The offspring conveys its particular need by a more or less explicit expression. The parent, primed by maternal hormones, may react with a fixed action pattern to this elicitor, e.g., a rat assumes the nursing posture in response to its pups’ cry. In mammals with more flexible maternal behavior, the mother may vicariously experience the offspring’s distress and act appropriately to address the problem. This flexible responding, of course, relies more on experience than does responding to a releaser. Presumably, the mother experiences vicarious gratification when she succeeds in satisfying the offspring. Affect may be less involved, or even absent, in the case of invariant responses to releasers because there is no need for affective feedback to shape the response. Flexible maternal behavior also requires knowing how to adjust to the offspring’s developmental changes and growing competence. For example, monkey mothers initially prevent the offspring from wandering off, then they accompany the offspring when it leaves, and in the last stage the mothers allow the offspring to venture away but periodically retrieve it.

Considering real-life scenarios in this way, and conducting observational research to elucidate them, might substantially advance our understanding of social emotions. Ekman (in Darwin [20]) defined an emotion as a brief event, but affective experience is often sustained or repetitive and yet retains its basic properties.

## 9. Conclusions

Tinbergen advised ethologists to describe the ontogeny, phylogeny, function, and proximate mechanisms for any behavior with an evolved basis [115]. Applying this injunction to the study of emotion, we should analyze the various facets of each basic emotion in developmental, evolutionary, neuroendocrine, and functional terms. Such an inventory might provide a basis for understanding pathological variations, such as an abnormally low or high threshold for a given emotion. For example, excessive fear can manifest itself as phobia or post traumatic stress disorder (PTSD), with concomitant changes in the amygdala [116], and insufficient fear as recklessness. Many psychopathological processes seem to involve aberrations from normal emotional functioning, so our understanding of these processes might benefit from elucidation of normal emotional mechanisms. 

## References

[1] Kleinginna P.R., Kleinginna A.M. (1981). A categorized list of emotion definitions, with suggestions for a consensual definition. Motiv. Emotion.

[2] Lazarus R.S., Scherer K.R., Ekman P. (1984). Thoughts on the relations between emotion and cognition. Approaches to Emotion.

[3] Zajonc R.B., Scherer K.R., Ekman P. (1984). On primacy of affect. Approaches to Emotion.

[4] Krolak-Salmon P., Hénaff M.-A., Vighetto A., Bertrand O., Mauguière F. (2004). Early amygdala reaction to fear spreading in occipital, temporal, and frontal cortex: A depth electrode erp study in human. Neuron.

[5] Maslow A.H., Lowry R. (1968). Toward a Psychology of Being.

[6] Panksepp J. (1998). Affective Neuroscience: The Foundations of Human and Animal Emotions.

[7] Pugh G.E. (1977). The Biological Origin of Human Values.

[8] Frijda N.H. (1988). The laws of emotion. Am. Psychol..

[9] Manstead A.S.R., Baumeister R.F., Finkel E.J. (2010). Social psychology of emotion. Advanced Social Psychology: The State of the Science.

[10] Van Hooff J.A.R.A.M., Von Cranach M. (1976). The comparison of facial expression in man and higher primates. Methods of Inference from Animal to Human Behaviour.

[11] Butler A.B., Hodos W. (2005). Comparative Vertebrate Neuroanatomy: Evolution and Adaptation.

[12] West-Eberhard M.J. (2003). Developmental Plasticity and Evolution.

[13] Tracy J.L., Shariff A.F., Cheng J.T. (2010). A naturalist’s view of pride. Emot. Rev..

[14] Tracy J.L., Robins R.W. (2008). The nonverbal expression of pride: Evidence for cross-cultural recognition. J. Pers. Soc. Psychol..

[15] Weisfeld G.E., Dillon L.M. (2012). Applying the dominance hierarchy model to pride and shame, and related behaviors. J. Evolut. Psychol..

[16] Schjelderup-Ebbe T., Murchison C. (1935). Social behavior of birds. A Handbook of Social Psychology.

[17] Alexander R.D. (1961). Aggressiveness, territoriality, and sexual behavior in field crickets (orthoptera: Gryllidae). Behaviour.

[18] Izard C.E. (1977). Human Emotions.

[19] Tomkins S.S., Scherer K.R., Ekman P. (1984). Affect theory. Approaches to Emotions.

[20] Darwin C. (1998). The Expression of the Emotions in Man and Animals.

[21] Tangney J.P., Miller R.S., Flicker L., Barlow D.H. (1996). Are shame, guilt, and embarrassment distinct emotions?. J. Pers. Soc. Psychol..

[22] Tracy J.L., Robins R.W. (2007). The psychological structure of pride: A tale of two facets. J. Pers. Soc. Psychol..

[23] Tangney J.P., Stuewig J., Mashek D.J. (2007). Moral emotions and moral behavior. Annu. Rev. Psychol..

[24] Wong Y., Tsai J. (2007). Cultural models of shame and guilt. The Self-Conscious Emotions: Theory and Research.

[25] Weisfeld G.E., Weisfeld M.B. (2013). Does a humorous element characterize embarrassment?. Int. J. Humor Res..

[26] Lombardo M.V., Baron-Cohen S., Belmonte M.K., Chakrabarti B., Decety J., Cacioppo J.T. (2011). Neural endophenotypes of social behavior in autism. The Oxford Handbook of Social Neuroscience.

[27] Storey A.E., Walsh C.J., Quinton R.L., Wynne-Edwards K.E. (2000). Hormonal correlates of paternal responsiveness in new and expectant fathers. Evol. Hum. Behav..

[28] Mak G.K., Weiss S. (2010). Paternal recognition of adult offspring mediated by newly generated cns neurons. Nat. Neurosci..

[29] Taylor S.E., Saphire-Bernstein S., Seeman T.E. (2010). Are plasma oxytocin in women and plasma vasopressin in men biomarkers of distressed pair-bond relationships?. Psychol. Sci..

[30] Ellis L. (1986). Evidence of neuroandrogenic etiology of sex roles from a combined analysis of human, nonhuman primate and nonprimate mammalian studies. Pers. Indiv. Differ..

[31] Mazur A. (2005). Biosociology of Dominance and Deference.

[32] Almeida O.P., Yeap B.B., Hankey G.J., Jamrozik K., Flicker L. (2008). Low free testosterone concentration as a potentially treatable cause of depressive symptoms in older men. Arch. Gen. Psychiatry.

[33] Carré J.M., Campbell J.A., Lozoya E., Goetz S.M., Welker K.M. (2013). Changes in testosterone mediate the effect of winning on subsequent aggressive behaviour. Psychoneuroendocrino..

[34] Coste S.C., Murray S.E., Stenzel-Poore M.P. (2001). Animal models of crh excess and crh receptor deficiency display altered adaptations to stress. Peptides.

[35] Selye H. (1956). The Stress of Life.

[36] Carter S.C., Porges S.W., Decety J., Cacioppo J.T. (2011). The neurobiology of social bonding and attachment. The Oxford Handbook of Social Neuroscience.

[37] Kosfeld M., Heinrichs M., Zak P.J., Fischbacher U., Fehr E. (2005). Oxytocin increases trust in humans. Nature.

[38] Heinrichs M., von Dawans B., Domes G. (2009). Oxytocin, vasopressin, and human social behavior. Front. Neuroendocrinol..

[39] Thompson R., George K., Walton J., Orr S., Benson J. (2006). Sex-specific influences of vasopressin on human social communication. Proc. Natl. Acad. Sci. USA.

[40] Hokanson J.E., Willers K., Koropsak E. (1968). The modification of autonomic responses during aggressive interchange1. J. Pers..

[41] Taylor S.E., Klein L.C., Lewis B.P., Gruenewald T.L., Gurung R.A., Updegraff J.A. (2000). Biobehavioral responses to stress in females: Tend-and-befriend, not fight-or-flight. Psychol. Rev..

[42] Ekman P., Friesen W.V. (1971). Constants across cultures in the face and emotion. J. Pers. Soc. Psychol..

[43] Ekman P., Ekman P., Davidson R.J. (1994). All emotions are basic. The Nature of Emotion: Fundamental Questions.

[44] DeCatanzaro D. (1999). Motivation and Emotion: Evolutionary, Physiological, Developmental, and Social Perspectives.

[45] Weisfeld G.E., Segal N.L., Weisfeld G.E., Weisfeld C.C. (1997). Discrete emotions theory with specific reference to pride and shame. Unitinig Psychology and Biology: Integrative Perspectives on Human Development.

[46] James W. (1884). II.—What is an emotion?. Mind.

[47] Lange C.G., Dunlap D. (1885). The mechanism of the emotions. The Emotions.

[48] Rolls E.T. (1999). The Brain and Emotion.

[49] Panksepp J., Ekman P., Davidson R.J. (1994). The clearest physiological distintions between emotions will be found among the circuits of the brain. The Nature of Emotion: Fundamenal Questions.

[50] Cannon W.B. (1927). The James-Lange theory of emotions: A critical examination and an alternative theory. Am. J. Psychol..

[51] Bard P. (1928). A diencephalic mechanism for the expression of rage with special reference to the sympathetic nervous system. Am. J. Physiol..

[52] Cacioppo J.T., Berntson G.G., Larsen J.T., Poehlmann K.M., Ito T.A., Lewis R., Haviland-Jones J.M. (2000). The psychophysiology of emotion. The Handbook of Emotion.

[53] Cobos P., Sánchez M., García C., Nieves Vera M., Vila J. (2002). Revisiting the james *versus* cannon debate on emotion: Startle and autonomic modulation in patients with spinal cord injuries. Biol. Psychol..

[54] Hohmann G.W. (1966). Some effects of spinal cord lesions on experienced emotional feelings. Psychophysiology.

[55] Laird J.D. (1974). Self-attribution of emotion: The effects of expressive behavior on the quality of emotional experience. J. Pers. Soc. Psychol..

[56] Lanzetta J.T., Cartwright-Smith J., Kleck R.E. (1976). Effects of nonverbal dissimulation on emotional experience and autonomic arousal. J. Pers. Soc. Psychol..

[57] Buck R. (1980). Nonverbal behavior and the theory of emotion: The facial feedback hypothesis. J. Pers. Soc. Psychol..

[58] Keillor J.M., Barrett A.M., Crucian G.P., Kortenkamp S., Heilman K.M. (2002). Emotional experience and perception in the absence of facial feedback. J. Int. Neuropsychol. Soc..

[59] Calder A.J., Lawrence A.D., Keane J., Scott S.K., Owen A.M., Christoffels I., Young A.W. (2002). Reading the mind from eye gaze. Neuropsychologia.

[60] Smith M.C., Smith M.K., Ellgring H. (1996). Spontaneous and posed facial expression in parkinson’s disease. J. Int. Neuropsychol. Soc..

[61] Kalat J. (2013). Personal communication.

[62] LeDoux J.E. (1996). The Emotional Brain: The Mysterious Underpinnings of Emotional Life.

[63] MacLean P.D. (1990). The Triune Brain in Evolution: Role in Paleocerebral Functions.

[64] Jarrell T.W., Gentile C.G., Romanski L.M., McCabe P.M., Schneiderman N. (1987). Involvement of cortical and thalamic auditory regions in retention of differential bradycardiac conditioning to acoustic conditioned stimuli in rabbits. Brain Res..

[65] Damasio A. (2005). Descartes’ Error: Emotion, Reason, and the Human Brain.

[66] Dunn B.D., Dalgleish T., Lawrence A.D. (2006). The somatic marker hypothesis: A critical evaluation. Neurosci. Biobehav. Rev..

[67] Maia T.V., McClelland J.L. (2004). A reexamination of the evidence for the somatic marker hypothesis: What participants really know in the iowa gambling task. Proc. Natl. Acad. Sci. USA.

[68] Barrash J., Tranel D., Anderson S.W. (2000). Acquired personality disturbances associated with bilateral damage to the ventromedial prefrontal region. Dev. Neuropsychol..

[69] Fellows L.K., Farah M.J. (2005). Different underlying impairments in decision-making following ventromedial and dorsolateral frontal lobe damage in humans. Cereb. Cortex.

[70] Heims H., Critchley H., Dolan R., Mathias C., Cipolotti L. (2004). Social and motivational functioning is not critically dependent on feedback of autonomic responses: Neuropsychological evidence from patients with pure autonomic failure. Neuropsychologia.

[71] Panksepp J. (2003). Looking for spinoza: Joy, sorrow, and the feelign brain. Conscious. Emot..

[72] Liotti M., Tucker D.M., Davidson R.J., Hugdahl K. (1995). Emotion in asymmetric corticolimbic networks. Brain Asymmetry.

[73] Schachter S., Singer J.E. (1962). Cognitive, social, and physiological determinants of emotional state. Psychol. Rev..

[74] Lindquist K.A., Wager T.D., Kober H., Bliss-Moreau E., Barrett L.F. (2012). The brain basis of emotion: A meta-analytic review. Behav. Brain Sci..

[75] Dethier V.G., Clark B. (1962). To Know a Fly.

[76] Carroll S.B. (2005). Endless Forms Most Beautiful: The New Science of Evo Devo and the Making of the Animal Kingdom.

[77] Vytal K., Hamann S. (2010). Neuroimaging support for discrete neural correlates of basic emotions: A voxel-based meta-analysis. J. Cogn. Neurosci..

[78] Phan K.L., Wager T., Taylor S.F., Liberzon I. (2002). Functional neuroanatomy of emotion: A meta-analysis of emotion activation studies in pet and fmri. Neuroimage.

[79] Murphy F.C., Nimmo-Smith I., Lawrence A.D. (2003). Functional neuroanatomy of emotions: A meta-analysis. Cogn. Affect. Behav. Neurosci..

[80] Hamann S. (2012). Mapping discrete and dimensional emotions onto the brain: Controversies and consensus. Trends Cogn. Sci..

[81] Bartels A., Zeki S. (2000). The neural basis of romantic love. Neuroreport.

[82] Coons E.E., Levak M., Miller N.E. (1965). Lateral hypothalamus: Learning of food-seeking response motivated by electrical stimulation. Science.

[83] Weaver D.R., Rivkees S.A., Reppert S.M. (1989). Localization and characterization of melatonin receptors in rodent brain by in vitro autoradiography. J. Neurosci..

[84] Zink C.F., Barter J.W., Decety J., Cacioppo J.T. (2011). Neural representation of social hierarchy. The Oxford Handbook of Social Neuroscience.

[85] Olds J., Milner P. (1954). Positive reinforcement produced by electrical stimulation of septal area and other regions of rat brain. J. Comp. Physiol. Psychol..

[86] Winkelman P., Berridge K., Sher S., Decety J., Cacioppo J.T. (2011). Emotion, consciousness, and social behavior. The Oxford Handbook of Social Neuroscience.

[87] King H.E., Sheer D.E. (1961). Psychological effects of excitation in the limbic system. Electrical Stimulation of the Brain.

[88] Gloor P. (1997). The Temporal Lobe and Limbic System.

[89] Black D.W. (1982). Pathological laughter: A review of the literature. J. Nerv. Ment. Dis..

[90] Martin J.P. (1950). Fits of laughter (sham mirth) in organic cerebral disease. Brain.

[91] Feinstein J.S., Adolphs R., Damasio A., Tranel D. (2011). The human amygdala and the induction and experience of fear. Curr. Biol..

[92] MacLean P.D., Lewis M., Haviland J.M. (1993). Cerebral evolution of emotion. Handbook of Emotion.

[93] LaFrenière P. (2010). Adaptive Origins: Evolution and Human Development.

[94] Sroufe L.A. (1997). Emotional Development: The Organization of Emotional Life in the Early Years.

[95] LaFreniere P.J. (2000). Emotional Development: A Biosocial Perspective.

[96] Buss K.A., Goldsmith H.H. (1998). Fear and anger regulation in infancy: Effects on the temporal dynamics of affective expression. Child Dev..

[97] Egas M., Riedl A. (2008). The economics of altruistic punishment and the maintenance of cooperation. Proc. R. Soc. Lond. B Biol. Sci..

[98] Trivers R.L. (1971). The evolution of reciprocal altruism. Q. Rev. Biol..

[99] Weisfeld G.E., Omark D.R., Strayer F.F., Freedman D.G. (1980). Social dominance and human motivation. Dominance Relations: An Etholgoical View of Human Conflict and Social Interaction.

[100] Harbaugh W.T., Mayr U., Burghart D.R. (2007). Neural responses to taxation and voluntary giving reveal motives for charitable donations. Science.

[101] Rilling J.K., Gutman D.A., Zeh T.R., Pagnoni G., Berns G.S., Kilts C.D. (2002). A neural basis for social cooperation. Neuron.

[102] De Quervain D.J.-F., Fischbacher U., Treyer V., Schellhammer M., Schnyder U., Buck A., Fehr E. (2004). The neural basis of altruistic punishment. Science.

[103] Averill J.R. (1983). Studies on anger and aggression. Am. Psychol..

[104] Fehr E., Gächter S. (2002). Altruistic punishment in humans. Nature.

[105] Pastore N. (1952). The role of arbitrariness in the frustration-aggression hypothesis. J. Abnor. Soc. Psych..

[106] Seip E.C., van Dijk W.W., Rotteveel M. (2009). On hotheads and dirty harries. Ann. NY Acad. Sci..

[107] Singer T., Seymour B., O’Doherty J.P., Stephan K.E., Dolan R.J., Frith C.D. (2006). Empathic neural responses are modulated by the perceived fairness of others. Nature.

[108] Ohbuchi K.-I., Kameda M., Agarie N. (1989). Apology as aggression control: Its role in mediating appraisal of and response to harm. J. Pers. Soc. Psychol..

[109] De Oliveira-Souza R., Hare R.D., Bramati I.E., Garrido G.J., Azevedo Ignácio F., Tovar-Moll F., Moll J. (2008). Psychopathy as a disorder of the moral brain: Fronto-temporo-limbic grey matter reductions demonstrated by voxel-based morphometry. Neuroimage.

[110] Beer J.S., Decety J., Cacioppo J.T. (2011). Neural systems of intrapersonal and interpersonal self-esteem maintenance. The Oxford Handbook of Social Neuroscience.

[111] Krajbich I., Adolphs R., Tranel D., Denburg N.L., Camerer C.F. (2009). Economic games quantify diminished sense of guilt in patients with damage to the prefrontal cortex. J. Neurosci..

[112] Barbey A.K., Grafman J., Decety J., Cacioppo J.T. (2011). The prefrontal cortex and goal-directed social behavior. The Oxford Handbook of Social Neuroscience.

[113] Weisfeld G.E. (2006). Humor appreciation as an adaptive esthetic emotion. Humor.

[114] Weisfeld G.E. (1993). The adaptive value of humor and laughter. Ethol. Sociobiol..

[115] Tinbergen N. (1951). The Study of Instinct.

[116] Rausch S.L., Shin L.M., Wright C.I. (2003). Neuroimaging studies of amygdala functino in anxiety disorders. Ann. NY Acad. Sci..

